# Workplace Bullying and Medically Certified Sickness Absence: Direction of Associations and the Moderating Role of Leader Behavior

**DOI:** 10.3389/fpsyg.2019.00767

**Published:** 2019-04-05

**Authors:** Morten Birkeland Nielsen, Anne-Marthe Rustad Indregard, Line Krane, Stein Knardahl

**Affiliations:** National Institute of Occupational Health, Oslo, Norway

**Keywords:** aggression, work ability, social support, justice, occupational health

## Abstract

The aim of this study was to determine (1) associations between workplace bullying and subsequent risk and duration of medically certified sickness absence, (2) whether employees’ perceptions of supportive, fair, and empowering leader behavior moderate the association between bullying and absence, and (3) whether prior sickness absence increases the risk of being a new victim of bullying. Altogether, 10,691 employees were recruited from 96 Norwegian organizations in the period 2004–2014. The study design was prospective with workplace bullying and leader behavior measured at baseline and then linked to official registry data on medically certified sickness absence for the year following the survey assessment. For analyses of reverse associations, exposure to bullying was reassessed in a follow-up survey after 24 months. The findings showed that workplace bullying was significantly associated with risk (risk ratio = 1.23; 95% CI = 1.13–1.34), but not duration (incidence rate ratio = 1.05; 95% CI = 0.89–1.25) of medically certified sickness absence after adjusting for age, gender, and supportive, fair, and empowering leader behavior. None of the indicators of leader behavior moderated the association between bullying and sickness absence (both risk and duration). Adjusting for baseline bullying, age, and gender, prior long-term sickness absence (>21 days) was associated with increased risk of being a new victim of bullying at follow-up (odds ratio = 1.86; 95% CI = 1.28–2.72). Effective interventions toward workplace bullying may be beneficial with regard to reducing sickness absence rates. Organizations should be aware that long-term sickness absence might be a social stigma as sick-listed employees have an increased risk of being bullied when they return to work.

During the last three decades, workplace bullying has been recognized as a highly important social stressor in both research and legislation (Samnani and Singh, 2012). Being defined as a situation where an employee is persistently and systematically exposed to harassment at work and where the employee finds it difficult to defend himself or herself against the harassment (Einarsen et al., 2011), bullying represents a form of long-lasting mistreatment. Hence, workplace bullying is not about single episodes of conflict or harassment at the workplace, but a form of persistent abuse where the exposed employee is unable to withstand or cope with the mistreatment (Einarsen, 1999; Einarsen et al., 2011). Both longitudinal studies and meta-analyses have established workplace bullying as a significant risk factor for health outcomes such as anxiety and depression (Nielsen and Einarsen, 2012; Verkuil et al., 2015), somatic complaints (Hoobler et al., 2010; Kääriä et al., 2012; Tynes et al., 2013), cardiovascular disease (Xu et al., 2018a,b), diabetes (Xu et al., 2018a,b), and disability retirement (Nielsen et al., 2017b). Comparisons with other psychosocial exposures show that bullying is one of the most detrimental predictors of health problems (Schutte et al., 2014).

While bullying has also been established as a predictor of sickness absence (Kivimäki et al., 2000; Niedhammer et al., 2013; Eriksen et al., 2016), existing research on this association has some important limitations: (1) The majority of studies have only examined direct association from workplace bullying to sickness absence (e.g., Kivimäki et al., 2000; Ortega et al., 2011). Consequently, with some notable exceptions (e.g., Nabe-Nielsen et al., 2016; Grynderup et al., 2017; Magee et al., 2017), little is known about the mechanisms and conditions that govern the association between the variables. (2) As shown in a review by Nielsen et al. (2016a,b), most studies have only examined the risk of having sickness absence without taking the duration of the absence period into account. (3) The potential impact of sickness absence on subsequent risk of bullying has largely been ignored, and it is to this date not established whether being sick-listed can be a risk factor for later exposure to workplace bullying.

With survey data from an extensive sample of Norwegian employees that has been linked to official registry data on medically certified sickness absence, the current study will contribute to fill these knowledge gaps. First, we will examine whether bullying influence both the risk of sickness absence (i.e., having at least 1 day with medically certified sickness absence within the year following the survey measurement) and duration of sickness absence (i.e., the number of days absent among those having absence). Second, we will determine the moderating impact of the behavior of the immediate leader on the association between bullying and sickness absence. Third, we will investigate the potential reverse impact of sickness absence on workplace bullying. In the following sections, we will elaborate these associations and propose our study hypotheses.

## Bullying and Sickness Absence

Taking into consideration that exposure to bullying, as a workplace stressor, is strongly associated with an increased risk of developing both mental and somatic health complaints (Nielsen et al., 2014; Verkuil et al., 2015), it is not surprising that bullying has been established as a robust predictor of sickness absence. Examining relations between 13 different psychosocial work factors and sickness absence in 31 European countries, Niedhammer et al. (2013) found that workplace bullying was the strongest psychosocial predictor of the risk of sickness absence. Reviewing all published research on workplace bullying and sickness absence, Nielsen et al. (2016a,b) found that exposure to bullying was associated with increased risk of having sickness absence in 94% of the included studies. A meta-analytic estimate of the association showed that targets of bullying had 1.58 higher odds (95% CI = 1.39–1.79) of exhibiting sickness absence compared to non-targets. While the evidence for an association between exposure to bullying and the risk of having sickness absence is robust, only Niedhammer et al. (2013) have examined whether bullying influences the length/duration of sickness absence. Their findings showed that exposure to bullying increased the duration of the absence among women, but not among men. However, due to the strong effects of bullying on both subsequent health complaints (Kivimäki et al., 2003; Finne et al., 2011; Xu et al., 2018a,b) and risk of disability retirement (Nielsen et al., 2017b), an association between bullying and the duration of absence seems reasonable. As a replication and extension of previous research on workplace bullying and sickness absence, this study will examine the impact of exposure to bullying on both the risk and the duration of sickness absence. The following hypothesis will be tested:

H1: Exposure to workplace bullying is associated with both an increased risk and an increased duration of sickness absence.

## The Protective Role of Leader Behavior

Although workplace bullying has been firmly established as a precursor to sickness absence, it is unlikely that all those exposed will respond to bullying in the same way, and some targets may be more prone to sickness absence compared to others (Nielsen et al., 2016a,b). While few studies have examined this notion about boundary conditions governing the relation between bullying and sickness absence empirically, there are theoretical reasons for expecting that the risk of absence following bullying should vary among targets due to different protective factors (Nielsen and Einarsen, 2018). According to most theories relating to work exposures and health, including the transactional model of stress and coping (Lazarus and Folkman, 1984), the demand-control (support) model (Karasek, 1979; Karasek and Theorell, 1990), the job demand resources model (Bakker and Demerouti, 2007), and the cognitive activation theory of stress (Ursin and Eriksen, 2004; Reme et al., 2008), the ability to withstand challenges at work is influenced by a range of individual and organizational coping possibilities and resources. These coping resources could be located at the level of the organization at large, at the interpersonal level, and at the individual level. With regard to handling the impact of workplace bullying, previous research has shown that resources at the interpersonal level may be more beneficial than resources at the individual level (Nielsen and Einarsen, 2018). Such interpersonal level resources include factors such as leadership, supervisor and coworkers’ support, and team climate (Bakker et al., 2003). It has been suggested that leadership may be an especially important protective factor regarding the consequences of workplace bullying (Stouten et al., 2010; Laschinger and Fida, 2014; Nielsen et al., 2016a,b).

Through controlling resources and by being central decision-makers, leaders can have a substantial impact on how subordinates experience their job and working conditions and thereby influence the well-being of the employee. Reflecting the impact on the organization and the employees, one may distinguish between constructive and destructive forms of leadership (Einarsen et al., 2007). Constructive leaders “adhere to the legitimate interests of the organization and support and enhance the organization’s goals, tasks, and strategy as well as making optimal use of organizational resources” (Einarsen et al., 2007, p. 214). They also increase the motivation, well-being, and job satisfaction of their subordinates through fostering extended engagement and by involving followers in decision processes (Einarsen et al., 2007; Aasland et al., 2010). While destructive forms of leadership should increase the risk of bullying (Hoel et al., 2010) and health problems (Skogstad et al., 2017), constructive forms of leadership contribute to reduce the occurrence of bullying (Nielsen, 2013) and should thereby also maintain health and well-being of their subordinates. In cases where bullying do occur, ideally constructive leaders should be able to deal with the bullying and help those exposed and thus buffer the impact of bullying on health outcomes. Building on this line of reasoning, we will examine whether three different forms of constructive leader behavior, that is supportive, fair, and empowering leadership, moderate the impact of workplace bullying on both the risk and the duration of sickness absence.

According to Dallner et al. (2000), *supportive leadership* reflects the degree to which the employee perceives the immediate leader as a source of social support and appreciation, and Podsakoff et al. (1990) defined supportive leadership as behavior on the part of the leader, which indicates that he or she respects his or her followers and is concerned with followers’ feeling and needs. A supportive leader focuses on relationships, shows commitment to the team members, and is attentive to the health and well-being of a subordinate. The subordinate will get the necessary support and help from the superior when needed, also in challenging situations. *Fair leadership* refers to the workers’ perceptions of the procedural justice or fairness in the decision-making process mediated by the superior. A fair leader treats their employees with respect and distributes work tasks and resources in a just manner. It has been suggested that perceived fairness of a leader influences levels of the working conditions of employees (Nielsen et al., 2018). *Empowering leadership* refers to the perceptions of the supervisor’s ability to encourage the employees to express their opinions and to develop themselves. This is achieved through fostering participation in decision-making process, providing autonomy, and making employees understand the purpose, goals, and objectives of the work. Hence, empowering leadership deals with arranging the distribution and exercise of power (Vecchio et al., 2010).

According to Stouten et al. (2010), leaders who encourage a positive work environment, and more specifically, by communicating what is appropriate and ethical behavior, should be able to reduce bullying as well as its impact on employees. Supportive and fair leader behavior have previously been associated with decreased risk of reporting bullying (Hauge et al., 2011), whereas all three types of leader behavior have been associated with good employee health (Christensen and Knardahl, 2014; Finne et al., 2014; Birkeland et al., 2016). However, with regard to risk of sickness absence following bullying, we expect that these forms of leader behavior have a differential impact due to how they approach employee’s well-being. By being attentive to the employee’s feelings, needs, and well-being, the supportive leader is likely to help the employee deals with the bullying and also try to maintain the health and well-being of the employee. Hence, leader support could be beneficial with regard to reducing the risk of sickness absence as this kind of leader behavior should decrease bullying and also the detrimental health outcomes following the exposure. Fair leadership should also be favorable with regard to reducing the risk of sickness absence following exposure to bullying as previous research have shown that perceptions of fairness play a crucial role in the experience of workplace bullying as well as for impact of bullying on the health and well-being of those exposed (Adoric and Kvartuc, 2007; Parzefall and Salin, 2010; Okechukwu et al., 2014). Hence, as bullying represents a serious deficiency in perceived organizational justice and fairness (Kivimäki et al., 2003), a fair leader is likely to react to the mistreatment and should be attentive to ways in which the situation can be resolved in a just and respectful manner. Through such actions, a fair leader should help the target to restore perceptions of justice and thereby reduce the risk for prolonged health problems and sickness absence following the bullying.

Although empowering leadership previously has been associated with good health and well-being (Peterson et al., 2008; Birkeland et al., 2017) and thereby on risk of sickness absence in general, we do not expect this form of leadership to protect against sickness absence following bullying. As empowering leaders promote the development of independence and autonomy of the subordinate, they are likely to facilitate the use of individual level resources and thereby encourage employees to solve problems by themselves. Previous research has shown that individual resources and power only have a protective effect in cases of no or only low exposure to bullying (Hewett et al., 2016; Nielsen et al., 2017c). In cases of high exposure, targets report equally high levels of health complaints irrespectively of their individual predispositions (Nielsen and Einarsen, 2018). Consequently, this kind of leader behavior is not likely to reduce the impact of workplace bullying on health and well-being and should therefore not have any moderating impact on risk of sickness absence due to bullying. To determine the impact of supportive, fair, and empowering leader behavior on risk of sickness absence following exposure to workplace bullying, the following hypothesis will be tested:

H2: Targets will have a lower risk of sickness absence following exposure to bullying if they perceive the immediate leader as supportive or fair, but not if they perceive the leader as empowering.

## The Impact of Previous Sickness Absence on the Risk of Bullying

Sickness absence is in itself a main precursor for future sickness absence, unemployment, work termination, and disability pension (Knapstad et al., 2014). Sickness absence can therefore lead to a deprivation of an important social arena, with social marginalization, isolation, and exclusion as possible results (Knapstad et al., 2014), all of which are related to workplace bullying (Einarsen et al., 2011). Supporting the potential effects of sickness absence on social interactions at the workplace, findings from a population-based survey of Swedish employees showed that previous sickness absence was associated with low perceived social support at work (Knapstad et al., 2014). Following the “behavioral mechanism” (Nielsen et al., 2017a), the potential impact of sickness absence on risk of bullying can be explained by irritation and anger in coworkers due to the practical consequences of sickness absence, that is colleagues have to do the job of the absent worker. Hence, in this perspective, having workers on long-term absence is likely to increase work load on colleagues who remains at work. This increased work load may be experienced as frustrating and may thereby trgger aggression. Group process theory may provide a similar explanation. Working in groups provides feelings of unity and secures a collective sense of identity for their members (Brown, 2000). However, a common finding in groups is that when a member of a group breaches expectations or group norms, other members are likely to reject the deviant (Festinger, 1950; Hutchison et al., 2007). Long-term or frequent sickness absence can be considered as a violation of group norms and thereby a form of deviance. In extreme cases, expelling the person from the group altogether through bullying and harassment can be a way of dealing with the undesirable member. Supporting this line of reasoning, previous research has shown that ill-health is a precursor for bullying and social exclusion (Finne et al., 2011; Nielsen et al., 2014). Consequently, we propose that employees who have had long-term sickness absence also have an increased risk of being exposed to bullying at work, and we will therefore elucidate that sickness absence is a potential risk factor for bullying in this study. The following hypothesis will be tested:

H3: Long spells of sickness absence is associated with an increased risk of subsequent victimization from bullying.

Taken together, we expect that bullying is associated with both the risk and the duration of sickness absence and, further, that leader behavior moderates this association. However, we suggest a differential impact of the investigated forms of leader behavior as only supportive and fair, but not empowering, behavior are expected to function as moderators. Finally, we expect that previous sickness absence is a predictor of bullying in that employees with long-term sickness absence are more likely to become bullied than employees without absence.

## Methods

### Design and Study Sample

The current study was an extension of the research project: “The new work place: Work, health, and participation in the new work life,” a longitudinal web-based survey carried out by the National Institute of Occupational Health (see Christensen and Knardahl, 2010; Finne et al., 2014; Emberland and Knardahl, 2015). The study design was prospective (organization were required to participate at least twice in the survey), with survey data linked to official registry data on sickness absence. This is an ongoing project with continuous gathering of survey data. While the organizations have participated at different dates, the time lags between the survey assessment points were equal for all respondents as the average time-period from the end of baseline survey to the end of follow-up survey was about 24 months. For a more detailed description of the research project, see the previously published study protocol (Nielsen et al., 2016a,b). This study is based on data from the baseline survey (Time 1), the first follow-up survey (Time 2), and the official registry data.

Some organizations were contacted by the National Institute of Occupational Health (NIOH) and offered to participate in the study, whereas other organizations contacted NIOH themselves in order to participate in the study. Recruitment and data collection took place from November 2004 to December 2014. After information about the general study aims was given at the organizational level, each employee, excluding those on sick leave, received a letter containing information about the survey, the strict confidentiality guidelines, and information about the license for data collection granted by the Norwegian Data Inspectorate. Each employee received a unique access code to the web-based questionnaire. A paper version of the questionnaire was sent out if requested in advance, but 85% of the baseline sample responded to the survey using the electronic survey form. The organizations represented a wide range of occupational sectors including healthcare, education, government and public administration, engineering, business, and industry. A detailed description of the recruitment has been published elsewhere (Christensen and Knardahl, 2010).

**Figure 1:**
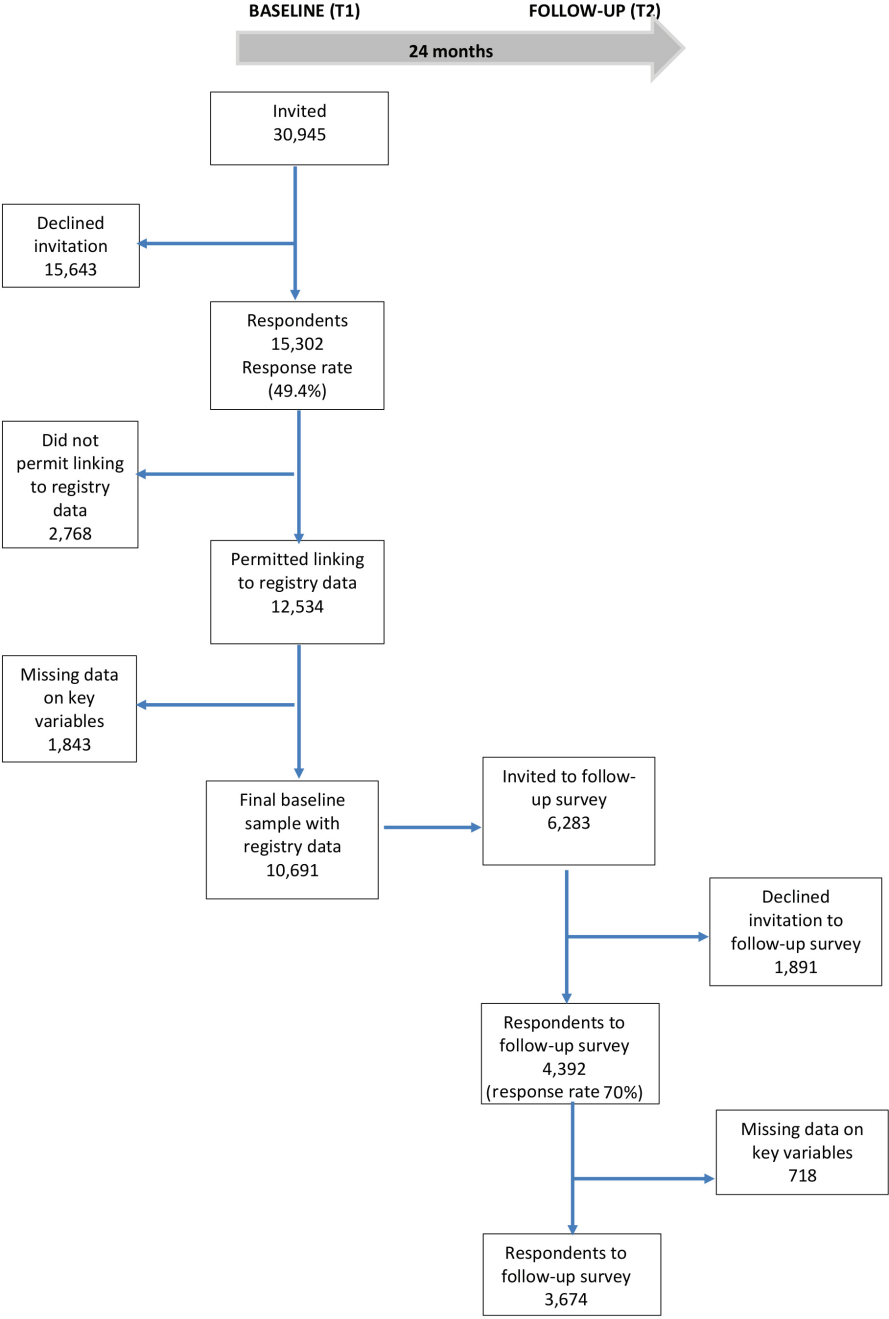
Flow chart for respondents.

A total of 30,945 adult employees in a full time or part time position, from 96 organizations, have so far been invited to participate in the baseline survey. Altogether, 15,302 persons have responded (response rate: 49.4%). Of these, 12,534 (82%) respondents permitted linking survey data to registry data on sickness absence. As 1,843 persons had missing data on study variables (i.e., not responded to the question about bullying or more than 25% missing on the questions about leadership), the final baseline sample comprised 10,691 respondents. Of the Time 1 respondents, altogether 6,283 persons have so far been invited to participate in the Time 2 survey, with 4,392 responding (70%). Remaining persons from the Time 1 survey have not participated in the follow-up survey because either: a) they had left their job between survey points/their employer did not wish to participate in the follow-up survey (altogether 4,782 persons) or b) their responses to the follow-up survey have not yet been included in the current dataset used in this study (357 persons). Hence, the attrition due to non-response from baseline to follow-up is 1,891 persons. Figure 1 presents a flow chart for the respondents.

The study sample consisted of more women (59.7%) than men (40.3%), and the mean age was 42.7 [standard deviation (SD) 10.59]. About 52% had minimum 13 years of education, 82.4% were permanently employed, and the majority did not have management responsibilities (82.6%). Occupations were classified according to the standard classification of occupations developed by Statistics Norway (STYRK; http://www.ssb.no), based on the International Standard Classification of Occupations (ISCO-88). The three largest occupational groups among all employees were *s*ervice workers and shop and market sales workers (28.5%), technicians and associate professionals (27.3%), and professionals (24.8%).

### Sickness Absence

We accessed information on medically certified sickness absence from the Norwegian Labour and Welfare Administration (NAV). The registry provides complete registrations of all medically certified sickness absence from the first day absent, including the length and medical diagnosis. The registry should be accurate since correct registration is required for the transfer of payments by the social insurance scheme. We aggregated data on sickness absence over 12- and 24-month follow-up post survey, which is consistent with previous research (Diestel and Schmidt, 2010; Nguyen et al., 2013). Registry information of sickness absence was linked to the survey data by the unique 11-digit national individual identity number. The time-period the employees were eligible for sickness absence was considered the same for all respondents within each company, starting from the day the electronic forms were closed. The registry was checked for inconsistencies. Overlapping or duplicate spells of sickness absence were merged.

### Workplace Bullying

Victimization from bullying was measured with a previously validated single-item question from the General Nordic Questionnaire for Psychological and Social Factors at Work (QPSNordic; Dallner et al., 2000). After being presented the following definition of workplace bullying: “Bullying and harassment (badgering, niggling, offending somebody) is a problem at some workplaces and for some workers. To label something bullying or harassment, the offensive behavior has to occur repeatedly over a period of time, and the person confronted has to experience difficulties defending himself/herself. The behavior is not bullying or harassment if two parties of approximately equal ‘strength’ are in conflict or the incident is an isolated event,” respondents were asked whether they had been subjected to bullying at the workplace during the last 6 months. The response categories were “yes” and “no.”

### Leader Behavior

Supportive, fair, and empowering leader behavior were measured by scales from the General Nordic Questionnaire for psychological and social factors at work (QPS Nordic) (Dallner et al., 2000; Wannstrom et al., 2009). Each scale contained three items asking about the behavior of the respondents’ immediate supervisor. Examples of the items include “If needed, can you get support and help with your work from your immediate superior?” (supportive leader behavior), “Does your immediate superior distribute the work fairly and impartially?” (fair leader behavior), and “Does your immediate superior encourage you to participate in important decisions?” (empowering leader behavior). The response categories ranged from “very seldom” (1) to very “often or always” (5). The internal consistencies (Cronbach’s alpha) for these scales were satisfactory (supportive leadership: 0.86; fair leadership: 0.85; empowering leadership: 0.87).

### Covariates

Covariates included in the multivariable models were selected on the basis of past research (Allebeck and Mastekaasa, 2004; Duijts et al., 2007). The variables included were gender and age (measured continuously in years).

### Statistical Analyses

The number of sickness absence days represents a form of count data, and Poisson regression is commonly used to analyze this outcome (North et al., 1993, 1996; Marmot et al., 1995; Kivimaki et al., 2001; Melchior et al., 2003; Rugulies et al., 2007). However, Poisson regression requires that the variance is equal to the mean, whereas for sickness absence data the variance is frequently substantially larger than the mean, a condition known as overdispersion (Cameron and Trivedi, 1998). Second, the number of events should follow the Poisson distribution, but the distribution of sickness absence often include more values of zeros (i.e., no sickness absence) than expected from the Poisson distribution. Ignoring overdispersion and excess of zero values may lead to a model with poor fit to the data, and tests of statistical significance will be unreliable (Christensen et al., 2007). In this study, we used a modified model for count data, the Negative binomial hurdle (NBH) model, which is capable of capturing both overdispersion and excess of zero values (Mullahy, 1986). The NBH model suggests a two-part process: (1) A log-binomial regression analysis, which estimated the risk ratio of having at least 1 day of medically certified sickness absence, and (2) a zero-truncated negative binomial analysis, which produced incidence rate ratios for the number of days absent among the sub-sample having at least 1 day absent. Finally, all included work factors were studied as independent variables simultaneously and adjusted for covariates. Mean scores of leadership were included as continuous independent variables in both parts of the hurdle model.

Logistic regression analyses were used to determine the impact of prior sickness absence on stability adjusted risk of workplace bullying. All statistical analyses were performed using STATA 14.2 (StataCorp, 2015) and SPSS version 25 (IBM Corp. Released, 2018).

## Results

### Descriptive Findings

During the last 6 months prior to the survey, 6.1 percent of the sample reported being bullied at their workplace. Based on official registry data, 39% of the sample had at least 1 day of sickness absence during the 12 months following the survey. Average number of days with absence the year following the survey was 23.16 (*SD* = 62.91). With regard to risk of sickness absence following the survey, women (44%) had significantly (*x^2^* = 171.98; *df* = 1; *p* < 0.001) higher prevalence compared to men (32%) and those exposed to bullying (50.4%) had significantly (*x^2^* = 37.06; *df* = 1; *p* < 0.001) higher prevalence compared to non-exposed colleagues (38.5%). With regard to duration of absence, women (*M* = 28.82; *SD* = 70.42) reported significantly (*t* = −10.89; *df* = 11,543; *p* < 0.001) more days compared to men (*M* = 15.87; *SD* = 51.0), and targets of bullying (*M* = 32.25; *SD* = 72.06) reported significantly (*t* = −3.81; *df* = 10,921; *p* < 0.001) more days than non-targets (*M* = 22.65; *SD* = 62.33).

### The Impact of Bullying on Sickness Absence

Findings from the Negative Binomial Hurdle model on direct effects of bullying and leader behavior on subsequent medically certified sickness absence, adjusted for age and gender, are presented in Table 1. Female gender (RR = 1.36; 95% CI = 1.29–1.43) and workplace bullying (RR = 1.23; 95% CI = 1.13–1.34) were significantly associated with increased risk of sickness absence in the log-binomial regression. Empowering leader behavior (RR = 0.93; 95% CI = 0.90–0.97) was associated with decreased risk of sickness absence. The negative binomial regression testing associations with duration of sickness absence showed that female gender (IRR = 1.49; 95% CI = 1.35–1.63) was associated with more days of absence. Bullying and leadership factors were not significantly associated with number of absence days.

**Table 1:** Hurdle analysis of associations between workplace bullying and different forms of leadership as predictor variables and medically certified sickness absence as outcome variable, adjusted for age and gender (*N* = 10,691).

Predictor variables	Log-binomial regression for risk of absence		Negative binomial regression for duration of absence	
	RR	95% CI	IRR	95% CI
Gender (reference: male)	1.36^***^	1.29–1.43	1.49^**^	1.35–1.63
Age	0.99^*^	0.99–0.99	1.02^***^	1.01–1.03
Workplace bullying (reference: not bullied)	1.23^***^	1.13–1.34	1.05	0.89–1.25
Supportive leadership	0.99	0.95–1.03	0.94	0.88–1.01
Empowering leadership	0.93^***^	0.90–0.97	0.96	0.90–1.02
Fair leadership	0.98	0.95–1.03	1.02	0.95–1.10

^***^p < 0.001; ^**^ p < 0.01; ^*^ p < 0.05.

CI, confidence interval; RR, risk ratio; IRR, incidence rate ratio.

### The Protective Role of Leader Behavior

A series of interaction analyses were conducted to examine the moderating effects of supportive, fair, and empowering leader behavior on the association between bullying and sickness absence. Neither supportive (RR = 1.02; 95% CI = 0.95–1.09), fair (RR = 1.02; 95% CI = 0.95–1.10) nor empowering leader behavior (RR = 1.98; 95% CI = 0.91–1.05) moderated the association between bullying and risk of medically certified sickness absence. Similar findings were established for the interaction between the indicators of leadership and bullying with regard to the duration of the absence (supportive leader behavior: RR = 1.01; 95% CI = 0.88–1.16; fair leader behavior: RR = 1.04; 95% CI = 0.90–1.21; empowering leader behavior: RR = 0.97; 95% CI = 0.84–1.13). The potential interactions between bullying and gender with regard to risk and duration of sickness absence were also tested without any significant findings.

### The Impact of Previous Sickness Absence on the Risk of Bullying

A logistic regression analysis examined the impact of prior medically certified sickness absence on changes in exposure to workplace bullying over time (Table 2). To be able to establish new cases of bullying during the survey period, we predicted bullying at the follow-up assessment adjusted for the respondents’ prior exposure to bullying at the baseline survey. Hence, analyses predicted the risk of being bullied at follow-up among those respondents who were not bullied at baseline. As there was a 24-month time-lag between the baseline and follow survey, we measured sickness absence within the same time-frame. In order to determine whether both the risk of having absence and the length of the absence predicted bullying, the sickness absence variables were recoded into five categories: “No absence,” “Up to seven days of absence,” “Between 8 and 14 days of absence,” “15 to 21 days of absence,” and “More than 21 days of absence.” Adjusted for age (OR = 1.00; 95% CI = 1.00–1.00), gender (OR = 0.75; 95% CI = 0.54–1.05), and prior exposure to bullying (OR = 29.76; 95% CI = 21.00–42.17), only respondents with more than 21 days of sickness absence between baseline and follow-up had a significantly higher risk of being a new case of bullying at follow-up (OR = 1.86; 95% CI = 1.28–2.72). Shorter spells of absence were not significantly related to risk of being bullied.

**Table 2:** Logistic regression of associations between the risk of medically certified sickness absence during the previous 24 months as predictor variable and workplace bullying as outcome variable, adjusted for age, gender, and previous exposure to bullying (*N* = 3,674).

Predictor variables	Risk of bullying follow-up	
	OR	95% CI
Gender (reference: male)	0.75	0.53–1.05
Age	1.00	1.00–1.00
Bullied at baseline (reference: no)	29.76^***^	21.00–42.17
Prior sickness absence (reference: no absence):		
1–7 days of absence	1.10	0.61–1.97
8–14 days of absence	0.86	0.44–1.68
15–21 days of absence	1.21	0.60–2.46
More than 21 days of absence	1.86^***^	1.28–2.72

*** p < 0.001.

CI, confidence interval; OR, odds ratio.

## Discussion

Replicating previous research findings (Nielsen et al., 2016a,b), the results of this study confirmed that workplace bullying is a significant predictor of sickness absence. Extending previous findings, our results showed that bullying was associated with increased risk of having medically certified sickness absence, but not with the duration of the absence spells. Empowering, but not fair and supportive, leader behavior was associated with decreased risk of having sickness absence. Neither supportive, fair, nor empowering leader behavior moderated the association between workplace bullying and sickness absence. As previous research has ignored the potential reverse impact of sickness absence on risk of experience bullying, a novel finding was that previous medically certified sickness absence above 21 days was significantly associated with subsequent workplace bullying, even after adjusting for previous exposure to bullying. Hence, the findings indicate that sickness absence may be a potential risk factor for being exposed to bullying at the workplace.

The finding that workplace bullying was associated with increased risk of having sickness absence, but not the duration of the absence suggests that exposure to bullying is a significant contributor of medically certified sickness absence, whereas the duration of the absence seems to be determinant by other factors. The association between bullying and risk of having absence shows that bullying is a severe workplace stressor with major impact on those exposed. Taking into consideration that previous longitudinal research has established that bullying increases the risk of both mental and somatic complaints (Nielsen et al., 2014; Verkuil et al., 2015), one explanation for the association between bullying and the risk of absence may that sickness absence is a way of coping with both bullying and health complaints. That is, by leaving work, the targeted employee will be able to escape the exposure to bullying, while also dealing with health issues. However, following this line of reasoning, it is somewhat surprising that bullying was not associated with the duration of the sickness absence. As bullying in part represents a subjective experience that is associated with worrying, rumination, and sleep problems (Moreno-Jimenez et al., 2009; Lallukka et al., 2011), it is likely that bullying should influence those exposed also outside work. Consequently, it seems that the duration of absence has little to do with the exposure to bullying, but that it is rather determined by other factors. Previous research has shown that several specific work exposures may be especially important with regard to the duration of absence. A Danish study of occupational predictors of long-term sickness absence found that role conflict, low reward, and poor management quality were related to long-term absence among women, whereas demands for hiding emotions and high emotional demands predicted long-term sickness absence among men (Lund et al., 2005).

Another explanation for the insignificant association between bullying and the duration of sickness absence may be that targets of bullying go back to work in order to demonstrate their commitment and loyalty to the employer and to avoid being characterized as malingerers (Hoel et al., 2011). In some cases, personal guilt in the form of self-inflicted presence could be another factor preventing people from taking longer time off, even if they, from a medical perspective, would benefit from staying at home (Nielsen and Einarsen, 2012). Finally, it may be that some employees refrain from taking longer sick leave because they fear retaliation from colleagues or from a destructive leader.

As most previous studies on bullying and absence have examined sickness absence as a dichotomous outcome (i.e., absence vs. no absence), albeit using different cut-off criteria and time-periods for absence (Kivimäki et al., 2000; Vingård et al., 2005; Aagestad et al., 2014), the established association between bullying and the risk of absence is in line with previous findings. Taken together, the findings from both this study and from previous research seem to provide robust evidence for bullying as a risk factor for sickness absence. Hence, successful interventions against bullying at the workplace could therefore also reduce sickness absence rates. With regard to potential interventions against bullying and sickness absence, we followed the suggestions by Nielsen et al. (2016a,b) and examined how different leader behavior may influence the effect of bullying on absence.

Contrary to our expectations of about a protective effect of fair and supportive leader behavior, but in support of empowering leader behavior being non-effective, the findings showed that none of the indicators of leader behavior moderated the association between bullying and absence. These non-significant effects of leader behavior suggest that if bullying is allowed to escalate the immediate leader may find it difficult to intervene in an effective manner and one may need assistance from the HR department or external consultants to resolve the conflict. As our findings show that leader behavior does not buffer the impact of bullying on sickness absence, measures directed against workplace bullying should therefore focus on primary prevention and interventions that may have an impact on the occurrence and outcomes of bullying. Previous research findings show that factors such as psychosocial climate (Bond et al., 2010; Einarsen et al., 2018) and target-specific interventions (Hodgins et al., 2014; Escartin, 2016) may be especially beneficial with regard to reducing the detrimental effects of bullying.

Although the findings did not show any moderating effects of leader behavior, a noteworthy result was that empowering leader behavior was associated with a decreased risk of having sickness absence in general. Empowering leadership encourages employees to develop self-control and to act on their own. Empowering leadership is an approach to leadership that offers prescriptions to leaders for arranging the distribution and exercise of power (Vecchio et al., 2010). The established impact of empowering leader behavior on the risk of sickness absence suggests that leaders who give their subordinates autonomy and promote self-leadership may benefit from reduced sickness absence rates.

Previous research on the precursors of workplace bullying has mainly focused on impact of psychosocial work factors (Van den Brande et al., 2016), target personality (Nielsen et al., 2017d), and mental health (Nielsen et al., 2014; Verkuil et al., 2015), whereas sickness absence as a potential social stigma has not been investigated. In this study, we found that employees with prior medically certified sickness absence above 21 days had a 1.86 higher odds for being new victims of bullying after adjusting for previous exposure to bullying. Hence, this finding supports the claim that long-term sickness absence may result in social marginalization, isolation, and exclusion (Bryngelson, 2009; Knapstad et al., 2014). As discussed in the introduction of this paper, an explanation for this association may be that sickness absence imposes increased work demands on coworkers that in turn may instigate irritation and anger. Furthermore, long-term absence may lead to a form of social marginalization at the workplace where the employee becomes a prototypical member of the work group due to violations of expectations about the presence at the workplace. As prototypical group members per definition deviates from the in-group, such members may elicit a perceived threat and their position in the group is likely to be questioned (Steffens et al., 2017). Hence, as they fail to maintain the distinctiveness of the group, prototypical members are evaluated more negatively by the group than non-prototypical members and are more prone to be targets of harassment and exclusion (Pickett and Brewer, 2005).

## Methodological Strengths and Limitations

Using a combination of questionnaire survey- and objective registry data, this study determined associations between workplace bullying, leader behavior, and medically certified sickness absence in a large cohort of Norwegian employees from a range of different Norwegian organizations and industries. Variables were assessed using psychometrically sound measurement instruments. The survey had a response rate in correspondence with the estimated average for organizational surveys (Baruch and Holtom, 2008). While the sample was large, the non-random recruitment of participating organizations limits the external validity of the findings. However, there was probability sampling at the individual level as all employees in the participating organizations were invited to participate in the survey (Ilies et al., 2003).

Because the indicators of leader behavior and bullying were self-report measures, the findings may be influenced by problems that are common to self-report methodology, such as response set tendencies. However, as the items have been constructed with the aim of avoiding emotive content and social desirability bias, the measures of supportive, fair, and empowering leader behavior should be rather insensitive to respondents’ emotions or personality traits (Christensen and Knardahl, 2012). Workplace bullying was measured with a single-item self-labeling question. Despite important limitations with single-item measures, there are also multiple advantages, such as cost-efficiency, greater face validity, and the increased willingness of respondents to take the time to complete the questionnaire when the number of items is reduced (Nielsen and Knardahl, 2015, p. 144). Single-item measures can be reliable, as estimated by test-retest correlations (Littman et al., 2006), correlate strongly with multiple-item scales (Wanous et al., 1997), and can predict outcomes effectively (e.g., Nagy, 2002). The measurement method for bullying included in this study is a commonly used approach within research on bullying and is recognized as a trustworthy and sound assessment of victimization from workplace bullying (Dallner et al., 2000; Nielsen et al., 2011).

The data for this study were collected in Norway between 2004 and 2014. It should be noted that the national setting of the study could influence the findings. In 2008, most Western development countries entered a period of economic recession (Giorgi et al., 2015). It is well established that the financial situation of a country influences the health and workability of workers. In a review of the literature, Mucci et al. (2016) found that the economic crisis was an important stressor that had a negative impact on workers’ mental health. Most of the studies documented that a rise in unemployment, increased workload, staff reduction, and wages reduction were linked to an increased rate of mood disorders, anxiety, depression, dysthymia, and suicide. Hence, it is likely that the financial crisis also had an impact on the incidence of bullying and sickness absence rates. As the financial crisis had limited impact on the Norwegian economy, direct comparisons with findings from other studies should be done with caution.

## Conclusions and Implications

This study fills important gaps in the research literature on workplace bullying and sickness absence. First, we have showed that workplace bullying is associated with the risk, but not the duration, of absence. Hence, targets of bullying are more prone to have sickness absence compared to their non-bullied colleagues, but the length of the absence spells are not different between bullied and non-bullied. Second, we have showed that supportive, fair, and empowering leader behavior do not protect targets of bullying against sickness absence, although empowering leader behavior may be beneficial with regard to reducing absence rates in general. Future research should therefore examine other potential factors that can moderate the impact of bullying on absence. Third, we have established long-term sickness absence as a possible precursor to workplace bullying indicating that sickness absence may be a social stigma in the workplace. To better elaborate this relationship, future research should replicate this finding in other samples and with other time lags.

The results of this study have several implications for practice. The finding that bullying is associated with increased risk of sickness absence indicates, as noted previously, that interventions against bullying may also be beneficial with regard to reducing sickness absence rates. An additional finding was that empowering leader behavior might be favorable with regard to reducing absence rates at large. Since the behavior of leaders did not protect targets of bullying against sickness absence, measures directed against workplace bullying should focus on primary prevention and interventions. Bullying is a complex social phenomenon that can stem from a wide range of antecedents and develop through multiple pathways. Knowledge about the causal relationship between bullying and other variables is therefore highly important with regard to the development of theoretical models as well as for creating effective interventions against bullying (Nielsen and Einarsen, 2018). As having long-term sickness absence increased the risk of subsequent bullying, our findings suggest that sickness absence can be one potential cause of bullying. An implication of this finding is that organizations can reduce the risk of bullying by providing extra support to employees who have been out of work due to long-term absence.

## Ethics Statement

This project was approved by the Regional Committees for Medical and Health Research Ethics (REC) in Norway (REC South East) and The Norwegian Data Protection Authority and was conducted in accordance with the World Medical Association Declaration of Helsinki. All study participants provided their informed consent. Respondents were treated anonymously in the data analyses. Only respondents who permitted the linking of their answers to registries are included in this study.

## Author Contributions

SK and MN were responsible for data collection. A-MI prepared the data. MN and A-MI analyzed the data. MN had the main responsibility for writing the manuscript. All authors contributed to the idea development and writing of the study.

### Conflict of Interest Statement

The authors declare that the research was conducted in the absence of any commercial or financial relationships that could be construed as a potential conflict of interest.
